# Pain-Related White-Matter Changes Following Mild Traumatic Brain Injury: A Longitudinal Diffusion Tensor Imaging Pilot Study

**DOI:** 10.3390/diagnostics15050642

**Published:** 2025-03-06

**Authors:** Ho-Ching Yang, Tyler Nguyen, Fletcher A. White, Kelly M. Naugle, Yu-Chien Wu

**Affiliations:** 1 Department of Radiology and Imaging Sciences, Indiana University School of Medicine, Indianapolis, IN 46202, USA; 2 Department of Anesthesia, Indiana University School of Medicine, Indianapolis, IN 46202, USA; 3 Stark Neurosciences Research Institute, Indiana University School of Medicine, Indianapolis, IN 46202, USA; 4 Department of Kinesiology, School of Health and Human Sciences, Indiana University Indianapolis, Indianapolis, IN 46202, USA; 5 Weldon School of Biomedical Engineering, Purdue University, West Lafayette, IN 47906, USA

**Keywords:** mild traumatic brain injury, diffusion tensor imaging, post-traumatic headache, endogenous pain measure, psychological-related assessments

## Abstract

**Background:** This study used diffusion tensor imaging (DTI) to detect brain microstructural changes in participants with mild traumatic brain injury (mTBI) who experienced post-traumatic headaches, a common issue that affects quality of life and rehabilitation. Despite its prevalence, the mechanisms behind post-traumatic headache are not well understood. **Methods:** Participants were recruited from Level 1 trauma centers, and MRI scans, including T1-weighted anatomical imaging and DTI, were acquired 1 month post-injury. Advanced imaging techniques corrected artifacts and extracted diffusion tensor measures reflecting white-matter integrity. Pain sensitivity assays were collected at 1 and 6 months post-injury, including quantitative sensory testing and psychological assessments. **Results:** Significant aberrations in axial diffusivity in the forceps major were observed in mTBI participants (*n* = 12) compared to healthy controls (*n* = 10) 1 month post-injury (*p* = 0.02). Within the mTBI group, DTI metrics at 1 month were significantly associated with pain-related and psychological outcomes at 6 months. Statistical models revealed group differences in the right sagittal stratum (*p* < 0.01), left insula (*p* < 0.04), and left superior longitudinal fasciculus (*p* < 0.05). **Conclusions**: This study shows that DTI metrics at 1 month post-injury are sensitive to mTBI and predictive of chronic pain and psychological outcomes at 6 months.

## 1. Introduction

Post-traumatic headache (PTH) is a significant and enduring consequence of mild traumatic brain injury (mTBI), with a prevalence ranging from 30% to 90% in civilian adult populations [1,2,3]. It is defined as a secondary headache that develops within 7 days following the injury [4]. PTH is regarded as chronic when it continues for more than 3 months, with 18–22% lasting more than 1 year [5,6] and approximately 2–5% lifetime prevalence [7]. Therefore, effective intervention and treatment will benefit millions of affected individuals, if the underlying PTH pathogenesis can be clarified by identifying reliable biomarkers.

Considerable overlap exists between PTH and more common headache disorders, such as migraine. However, migraine-specific preventive medications are largely reported to be ineffective for PTH [8]. PTH mechanisms responsible for pathophysiology likely include abnormal endogenous pain modulation, activation of the somatosensory system, and psychological symptoms. Previous studies have demonstrated that mTBI produces sensitization of the head area (i.e., lower pressure pain thresholds (PPT) in the head region) and reduced pain inhibition during the conditioned pain modulation (CPM) test when compared to healthy controls [9]. Reduced pain-inhibitory capacity, soon after the injury, is a risk factor for the development of persistent PTH [9]. These results have been mirrored in pre-clinical studies demonstrating impaired descending noxious inhibitory controls and heightened sensitization to noxious mechanical stimuli in mice for a duration of up to 2 weeks after mTBI [10]. Further, multiple studies have reported increased anxiety, depression, and pain catastrophizing among patients with persistent PTH [9,11].

Diffuse axonal injury is also generally believed to be the initial neuropathology associated with mTBI [12,13,14]. A few studies report evidence of diffuse axonal injury in postmortem pathological examination of human mTBI. These papers suggest severing of nerve fibers without hemorrhage [15], the presence of amyloid precursor protein indicating axonal damage [16], and polarized macrophages in white matter [17]. Additionally, evidence of axonal injury in conjunction with chronic traumatic encephalopathy has been demonstrated in football players exposed to repetitive head impacts and/or concussion [18].

Nevertheless, this microscopic white-matter injury has been more difficult to detect in human mTBI with in vivo approaches. Neuroimaging may provide significant advancement for quantitatively detecting and characterizing the mechanisms of brain changes associated with pain-related outcomes following mTBI. While conventional diagnostic magnetic resonance imaging (MRI) presents negative findings in PTH after mTBI, recent studies have explored advanced imaging modalities to provide better diagnostic and prognostic utility [19,20]. For example, functional MRI has been widely used to explore both ascending and descending nociceptive pathways in the cortico-mesolimbic system for patients with chronic pain [21,22,23,24,25]. Also, susceptibility-weighted imaging (SWI) is capable of detecting localized microbleeds that are highly associated with axonal injury in mTBI [26,27], but it has not been used for PTH detection. Furthermore, arterial spin labeling has been used to assess cerebral perfusion impairment in mTBI. Despite inconsistent results, more findings indicate decreased rather than increased cerebral blood flow after mTBI [28,29]. With respect to PTH, perfusion-based functional connectivity of the insular subregion has been shown to be significantly associated with headache features [30], but interestingly, it did not show significant associations in female PTH at 2 weeks after pediatric concussion [31].

Diffusion MRI, however, may offer greater sensitivity in comparison with the imaging modalities mentioned above for detecting white-matter microstructure changes in mTBI [32,33,34]. The classic diffusion tensor imaging (DTI) has been widely used in white-matter diseases and was found to have adequate diagnostic sensitivity to microstructural changes in the brain after mTBI [35,36,37,38,39,40,41,42,43,44]. Despite its popularity, there have been few DTI studies focusing on PTH. In these few studies, scientists have observed white-matter differences between participants with and without PTH [45,46,47]. For example, patients with mTBI and PTH exhibited lower fractional anisotropy (FA) in the corpus callosum and fornix/septohippocampal circuit in comparison with healthy controls [48]. Similar findings of lower FA were found in the genu of the corpus callosum in mTBI patients with PTH compared to healthy controls by utilizing a principal component analysis [49]. A recent study investigated the association between white-matter structural connectivity and quantitative pain measurements but without providing further characteristics of white-matter microstructure [50]. While these studies provide insights into one DTI metric (i.e., FA) in PTH, they did not leverage the full potential of DTI by including all four metrics to encompass a holistic interpretation for microstructural changes in white matter in PTH. Moreover, previous studies (except [49]) did not include pain-related outcomes in the analyses. To address these knowledge gaps, this study will investigate all four DTI metrics, including FA, mean diffusivity (MD), radial diffusivity (RD), and axial diffusivity (AD), with a comprehensive survey of pain-related outcomes, including quantitative, psychological, and clinical pain assessments.

In this pilot study, we characterized white-matter microstructural alterations in mTBI patients with headache or at risk of developing headache. We studied (1) between-group differences (mTBI and control) at 1 month post-injury, (2) associations between early DTI metrics at 1 month post-injury and later pain- and psychological-related assessments at 6-month post-injury to examine the predictivity of DTI metrics in mTBI group, and (3) between-group (mTBI and control) prediction power differences for those significant associations within mTBI group.

## 2. Materials and Methods

### 2.1. Participants

The clinical coordinators screened electronic patient medical records for patients who had experienced mTBI and also met the associated inclusion and exclusion criteria. The mTBI diagnosis for each patient is outlined by the World Health Organization Task Force [51]. Next, potentially qualified mTBI patients’ identification and contact information were entered into a secure database for recruitment. Age- and sex-matched control participants with no TBI history were also recruited from the community, following the same exclusion criteria as outline below [52].

The inclusion criteria for the mTBI participants were level of consciousness, a clean CT test, experience of an altered mental status, and an adult under the age of 66. Secondly, the exclusion criteria included cardiovascular disorders, chronic systemic diseases, neurologic disorder, severe mental health disorder, previous chronic migraine, active legal proceedings, prolonged narcotic dependence, head injuries with fractural trauma, metallic implants, cognitive impairment, or pregnancy [52].

All mTBI participants received pain and psychological assessments at 1 month and 6 months post-injury [52]. A subset of the mTBI participants (*n* = 13) received MRI scans at 1 month post-injury and was included in this study to investigate the relationship and predictivity of neuroimaging and later pain-related outcomes. The controls (*n* = 10) completed two study sessions separated by 5–6 months and received the same pain and psychological assessments and neuroimaging.

### 2.2. Quantitative Pain Assessments

*Quantitative sensory tests* (QSTs) were used to measure pain sensitivity of the head and endogenous pain modulation. These tests included temporal summation of pain (TS) as an indirect method of assessing hyperexcitability of the central nervous system [53], PPTs measuring trigeminal sensitization [54], and conditioned pain modulation (CPM) assessing endogenous pain inhibition [55,56]. The TS test was performed first, followed by PPTs of the head, and then the CPM test. Before each session, all participants were asked to refrain from pain-relief medication and consuming caffeine on the day of testing.

*Mechanical Temporal Summation* (TS) was tested on the middle of the forehead and the back of the hand using a Von Frey filament (Touch test Sensory Evaluator 6.65, North Coast Medical, Inc., Morgan Hill, CA, USA) calibrated to bend at 300 g of pressure. First, a single pinprick with the filament was applied to the body site. Then, a series of 10 pinpricks with the same filament was applied to the same body site within an area of 1 cm^2^ and at a rate of 1 prick per second. Participants rated their pain after the single pinprick and after the series of 10 pinpricks using a 0–100 scale. TS was calculated as the difference between the single-pinprick pain-rating and the series of 10-pinpricks pain-rating. This TS procedure was administered twice with pauses at each location, then averaged for one forehead and hand scores [52].

*Pressure Pain Thresholds of the Head* (PPTs) were assessed by measuring sensory response on the head with an algometer. The probe was applied in two series, separated by a pause, with constant pressure against the skin on five sites. Participants alerted the examiner when first experiencing pain during the test. The scores from all trials were averaged to obtain one score for each participant [52].

*Conditioned Pain Modulation* (CPM) evaluates whether pain produced by a test stimulus is diminished by a second painful conditioning stimulus applied to a remote body site [55,56]. In this study, one hand was submerged in a cold-water bath (conditioning stimuli), and the PPTs (test stimuli) were immediately conducted on the opposite arm. This sequence was repeated for a second time. The magnitude of CPM reveals the amount of the altered-pain threshold following conditional stimuli [52].

### 2.3. Psychological and Clinical Pain Assessments

Head pain and psychological measures were collected via validated questionnaires as described below.

*Headache survey*: The participant survey was designed to demonstrate the severity of headache intensity (HA), including a range of related factors. Headache intensity was used for data analysis [52].

*McGill Pain Questionnaire* (MPQ) offers an objective level and description of the pain a participant is experiencing [57]. The pain-rating index (PRI) provides a descriptive ranking for the severity of a person’s associated pain.

*Defense and Veterans Pain Rating Scale* (DVPRS): 0 indicates “no pain”, and 10 indicates “as bad as it could be, nothing else matters” [58].

*TBI Quality of Life* (TBI-QOL) Headache Form assesses the severity and impact of headaches over the last 7 days with 10 items [59].

*Post-Traumatic Stress Disorder* (PTSD) for DSM-5 (PCL-5) is a 20-item scale that is designed to evaluate the DSM-5 symptoms of PTSD, and it is used for diagnosis and as a severity measure [60].

*Pain-Catastrophizing Scale* (PCS) uses the Likert scale to assess a participant’s mental condition, demonstrating the effect of negative thoughts resulting in actual pain. Higher scores indicate greater pain catastrophizing [61].

*Center for Epidemiological Studies—Depression Scale* (CES-D) is a self-report depression scale used to evaluate participant symptoms [62].

### 2.4. Image Acquisition

The participants underwent MRI scans in Siemens MAGNETOM Biography mMR 3T scanner (Siemens Medical Solutions USA, Inc., Malvern, PA, USA) with a 20-channel head/neck coil. The MRI scans included T1-weighted anatomical imaging and diffusion-weighted imaging (DWI). The total MRI examination time is 44 min and 38 s. The T1-weighted images were acquired by inversion recovery-prepared spoiled gradient-echo (IRSPGR) with TR/TE = 1960/2.19 ms, flip angle = 10 deg, and 1.0 × 1.0 × 1.0 mm^3^ voxels, with a scan time of 4 min and 34 s. Diffusion MRI was performed with a single-shot echo-planar imaging sequence and consisted of 64 directions at b value of 1000 s/mm^2^ and 1 b0 (b value = 0 s/mm^2^). Other imaging parameters were field-of-view = 2080 mm, 60 slices, voxel size = 2.0 × 2.0 × 2.0 mm^3^, TR/TE = 10,900/79 ms, in-plane GRAPPA acceleration factor = 2, with a scan time of 12 min and 21 s. For distortion correction, a dual-echo gradient-echo sequence (GRE) was used to acquire the field map with TR = 400 ms, TE = 4.92/7.38 ms, with a scan time of 54 s.

### 2.5. Diffusion MRI Data Analysis

The DWI were initially denoised with a Marchenko–Pastur principal component analysis approach [63] followed by Gibbs ringing artifacts removal using local subvoxel-shifts command in MRtrix3 (mrdegibbs; www.mrtrix.org) [64]. The motion, eddy current, and susceptibility artifacts correction were performed by using a dual-echo GRE field-map and T1-weighted image with the eddy_openmp command provided in the FMRIB Software Library (FSL; https://fsl.fmrib.ox.ac.uk/fsl/docs/#/) [65]. The FSL eddy command detects outlier slices using a Gaussian process prediction. B1 field inhomogeneity was also corrected (MRtrix3 dwibiascorrect) [66]. Four classic DTI metrics were calculated and registered to the Montreal Neurological Institute (MNI) template using a nonlinear registration tool, ANTs (Advanced Neuroimaging Tools) [67]. The DTI metrics included fractional anisotropy (FA), mean diffusivity (MD), radial diffusivity (RD), and axial diffusivity (AD).

**Figure 1:**
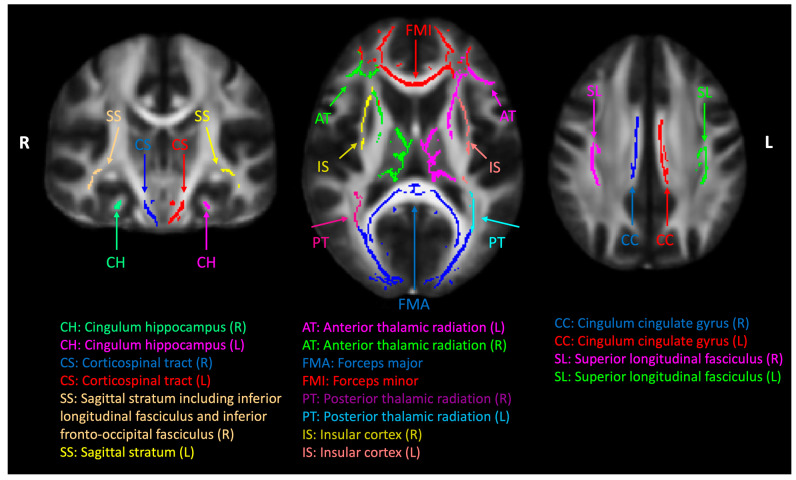
Cerebral white-matter tract regions of interest (ROIs). A common white-matter skeleton was first generated by the tract-based spatial statics (TBSS) toolbox in the FMRIB Software Library (FSL) using normalized diffusion tensor imaging (DTI) factional anisotropy maps from all the subjects. The white-matter skeleton was intercepted with Johns Hopkins University (JHU) white-matter tract atlas, and eighteen ROIs were used in this study.

A study-specific, whole-brain, white-matter skeleton was created using the FSL toolbox [68]. Eighteen bilateral regions-of-interest (ROIs) were segmented by intersecting the white-matter skeleton template and the JHU White-Matter Tractography Atlas [47,68,69] (Figure 1).

### 2.6. Statistical Analysis

The Kolmogorov–Smirnov test of normality indicated that most of the diffusion metrics, QSTs, psychological, and clinical measures followed a normal distribution in both groups (see Supplementary Tables S1 and S2 for details). However, normality was not observed in headache, MPQ, and TBIQL scores for the control group, as the majority of control participants had a score of 0 for headache (*n* = 9), MPQ (*n* = 9), and TBIQL (*n* = 8). In such cases, normality testing is not meaningful because a distribution with a large proportion of identical values (such as 0) is not continuous and will not follow a normal distribution. Thus, parametric tests were used to analyze these data. This study utilized a two-sample independent T-test to examine group differences in continuous variables related to demographic and clinical outcome measures, as presented in Table 1. Sex, as a categorical variable, was analyzed using $\chi^{2}$ tests to assess differences in sex compositions across groups. Generalized linear regression models were used to test (1) group differences in the DTI metrics between mTBI and control participants at 1 month, (2) associations of the early DTI metrics at 1 month with later outcome measures at 6 months to assess predictivity of DTI in mTBI, and (3) group differences in the predictivity of DTI for pain and psychological outcome measures. For the group differences in the DTI metrics, we used Model 1 controlling for age and sex, as well as the DVPRS pain rating, to minimize the effect of acute general pain and to focus on chronic headache at the time of MRI scans (Equation (1)). For the predictivity evaluation in the mTBI group, we used Model 2 controlling for age, sex, and the DVPRS scores (Equation (2)). Since there is a possibility of observing similar significant associations in control participants as well as those significant associations in mTBI participants in Model 2, we further tested whether there were group (mTBI and control) differences in the predictivity of DTI using Model 3 (Equation (3)), which included an interaction term of “group*DTI”. Specifically, if β_3_ in Model 3 was significant (i.e., *p*_int_ < 0.05), there would be significant group differences in the associations between outcomes_6-mo_ and DTI_1-mo_. When the interaction term is significant (i.e., *p*_int_ < 0.05), we performed post hoc correlation analyses in the control group using Model 2 and compare the scatterplots between the 2 groups. The significance threshold was set at *p* < 0.05 for all tests, with exact *p*-values for each measure reported in the Results Section. Due to the exploratory nature of this pilot study and small sample size, we did not adjust for multiple comparisons [70].

Model 1:

(1) $$DTImetrics={\beta_{0}+\beta }_{1}\cdot group+\beta_{2}\cdot age+{\;\beta }_{3}\cdot sex+\beta_{4}\cdot DVPRS+\varepsilon$$

Model 2:

(2) $${Outcomes}_{6mo}=\beta_{0}+{\;\beta }_{1}\cdot {DTI}_{1mo}+\beta_{2}\cdot age+\beta_{3}\cdot sex+\beta_{4}\cdot DVPRS+\varepsilon$$

Model 3:

(3) $$\begin{array}{cc} {Outcomes}_{6mo}= & \beta_{0}+\beta_{1}\cdot {DTI}_{1mo}+{{\;\beta }_{2}\cdot group+{\;\beta }_{3}\cdot group\cdot {DTI}_{1mo}+\beta }_{4}\cdot age\\ & +\beta_{5}\cdot sex+\beta_{6}\cdot DVPRS+\varepsilon \end{array}$$

## 3. Results

Thirteen mTBI and ten control participants received neuroimaging at 1 month post-injury. One mTBI participant’s MRI data were excluded due to an extended artifact from a dental implant, making the final sample size for the mTBI group twelve. The demographic characteristics of the participants are listed in Table 1. There were no significant differences between the mTBI and control participants in age (*p* = 0.45) and sex (*p* = 0.44). Compared to the controls, the mTBI participants had significantly higher scores on the PCL (*p* = 0.03) and CES-D (*p* = 0.04) at 1 month post-injury and higher scores in all the clinical pain measures (i.e., HA, MPQ, TBIQL, and DVPRS) (*p* < 0.01) at 1 month and 6 months post-injury. Moreover, it is noteworthy that our mTBI participants exhibited significant headache symptoms. Eleven mTBI participants reported experiencing new or worse headaches within 1 week of the mTBI, which were still present at 1 month post-injury. Nine of these mTBI participants still reported experiencing regular headaches at 6 months post-injury (data missing for 2 participants). The average headache-intensity score among the mTBI participants was significantly higher than the controls at 1 month post-injury (*p* = 0.0001) and at 6 months post-injury (*p* = 0.01).

Approximately half of the mTBI participants reported taking medications for their headaches. Specifically, at the 1 month post-injury visit, six participants reported taking medications for their headaches, including acetaminophen (*n* = 4), NSAIDS (*n* = 6), medicine that treats muscle spasms (*n* = 1), and anti-anxiety (*n* = 1) and antidepressant (*n* = 1) medicine. At the 6 months post-injury visit, seven participants reported taking medications for their headaches, including acetaminophen (*n* = 4), triptans (*n* = 1), NSAIDS (*n* = 4), and anti-anxiety (*n* = 1) and antidepressant (*n* = 1) medicine. All participants reported taking the medicine only when a headache was present (i.e., for abortive purposes). These medications were not taken on the day of testing, before the session took place.

Abbreviations: mild traumatic brain injury (mTBI); *p* = significance level; mo = month; TS = temporal summation of pain measuring endogenous facilitation of pain of the forehead areas; PPT = pressure pain threshold measuring sensitization of the head area; CPM = conditioned pain modulation measuring endogenous pain inhibition; PCS = pain catastrophizing scale; PCL = post-traumatic stress symptoms questionnaire; CES-D = Center for Epidemiological Studies—Depression scale; HA = headache intensity; MPQ = McGill Pain Questionnaire measuring different aspects of head pain in the past week; TBI-QOL = TBI Quality of Life Headache scale measuring subjective experience of headache symptoms for those with TBI; DVRS = Defense and Veterans Pain Rating Scale.; MRI = magnetic resonance imaging.

### 3.1. Group Differences in the DTI Metrics

**Figure 2:**
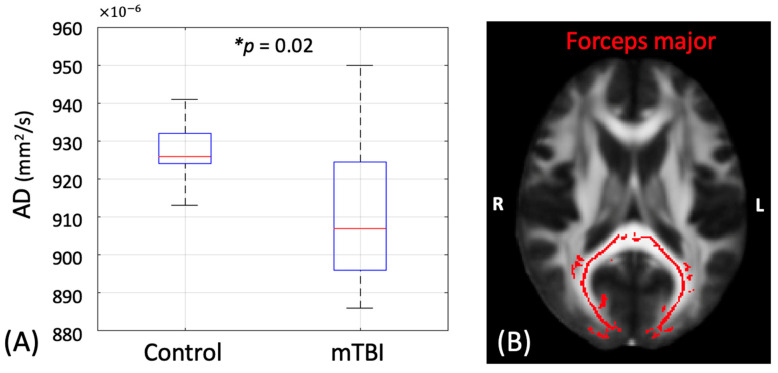
(**A**) Bar plot of DTI axial diffusivity (AD) between the mild traumatic brain injury (mTBI) participants and controls in the forceps major at 1 month post-injury. * denotes significant *p* value less than 0.05. (**B**) The forceps major fiber tract is highlighted in red. The image is presented in radiology orientation.

The mTBI participants exhibited significantly lower AD in the forceps major (*p* = 0.02) than the controls at 1 month post-injury (Figure 2). Other DTI metrics and white-matter fiber tracts did not differ significantly between groups.

### 3.2. Association Between the Early DTI Metrics and Later Pain- and Psychological-Related Measures

Within the mTBI group, the DTI metrics at 1 month post-injury had significant associations with the pain-related measures at 6 months post-injury (Table 2). The QST TS of the head was predicted by DTI FA in the right sagittal stratum, including the inferior longitudinal fasciculus and inferior frontal occipital fasciculus, with a large correlation coefficient, r = −0.60 (*p* = 0.01). The QST PPT was predicted by DTI AD in the left anterior thalamic radiation, corticospinal tract, and insula with a moderate correlation coefficient, r > 0.13 (*p* < 0.05). Furthermore, CPM had a significant positive association with FA in left superior longitudinal fasciculus (r = 0.83, *p* = 0.02).

The DTI metrics also predicted later psychological outcomes (Table 2, middle section). PCL and CES-D were predicted by both FA (negative association) and RD (positive association) in the right sagittal stratum with large correlation coefficients (|r| > 0.60, *p* < 0.05). They were also predicted by MD and RD in the left superior longitudinal fasciculus (positive association, r > 0.73, *p* < 0.05). PCS was predicted by MD in the left insular (positive association, r = 0.68, *p* = 0.03) and AD in the left corticospinal tract (positive association, r = 0.69, *p* = 0.04).

Regarding the clinical pain measures, the MPQ pain scale at 6 months also increased with elevated RD in the left corticospinal tract (r = 0.67, *p* = 0.01) at 1 month post-injury. All above associations had a median goodness-of-fit (R^2^) of 0.75 (ranging from 0.64 to 0.94).

### 3.3. Between Group Prediction Power Differences

**Figure 3:**
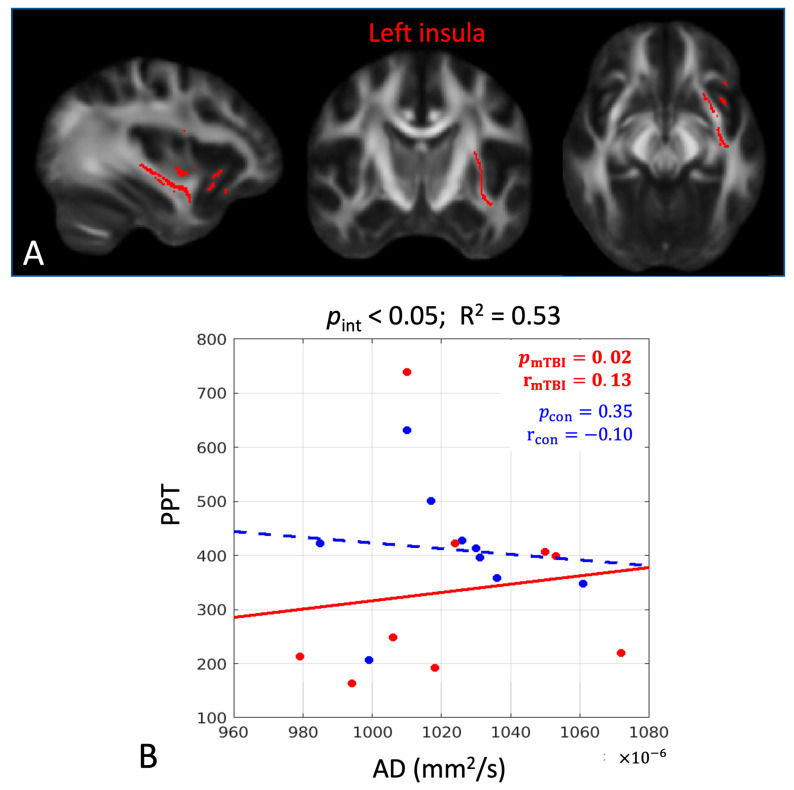
In the left insula, there was a significant group difference in the associations between axial diffusivity (AD) at 1 month post-injury and the pressure pain threshold (PPT) of the trigeminal sensitization at 6 months post-injury. (**A**) The significant white-matter tract in the left insula is shown in red. (**B**) Scatter plots with post hoc correlation results. The mTBI group had a significant positive association (red dots and solid line), while the control group did not (blue dots and dashed line). Abbreviations: *p*_int_ = significance level of the group interaction term in Equation (3); R^2^ = overall coefficient of determination for goodness of fit for Model 3; *p*_mTBI_ = significance level of the association for the mTBI group; r_mTBI_ = correlation coefficient for the mTBI group; *p*_con_ = significance level of the association for the control group; r_con_ = correlation coefficient for the control group. Bold numbers indicate statistical significance with *p* < 0.05.

**Figure 4:**
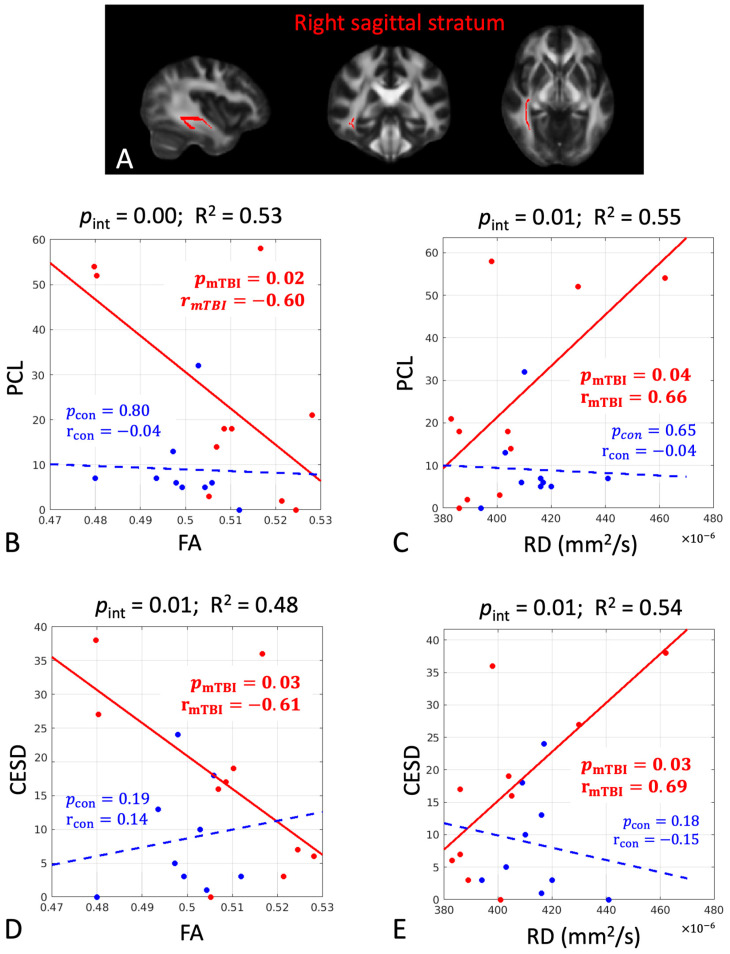
In the right sagittal stratum, there were significant group differences in the associations between the DTI metrics of fractional anisotropy (FA) and radial diffusivity (RD) at 1 month post-injury and the psychological measures of post-traumatic stress symptoms questionnaire score (PCL) and the depression scale at 6 months post-injury (CESD). (**A**) The significant white-matter tract is shown in red. (**B**–**E**) Scatter plots with post hoc correlation results. The mTBI group had a significant positive association (red dots and solid line), while the control group did not (blue dots and dashed line). Abbreviations: *p*_int_ = significance level of the group interaction term in Equation (3); R^2^ = overall coefficient of determination for goodness of fit for Model 3; *p*_mTBI_ = significance level of the association for the mTBI group; r_mTBI_ = correlation coefficient for the mTBI group; *p*_con_ = significance level of the association for the control group; r_con_ = correlation coefficient for the control group. Bold numbers indicate statistical significance with *p* < 0.05.

**Figure 5:**
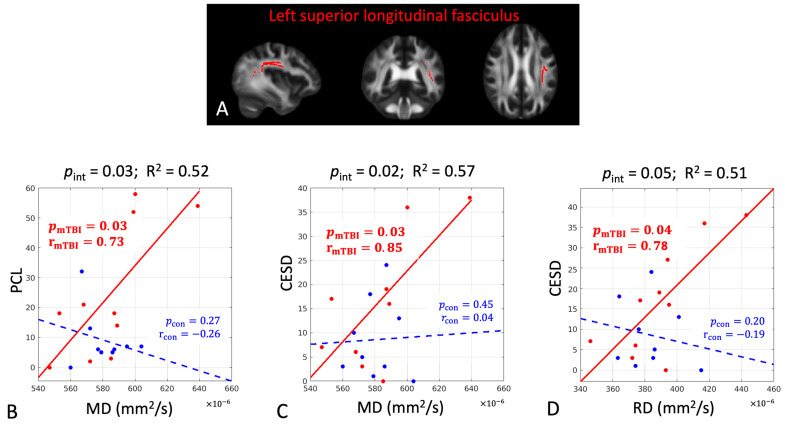
In the left superior longitudinal fasciculus, there were significant group differences in the associations between the DTI metrics of mean diffusivity (MD) and radial diffusivity (RD) at 1 month post-injury and the psychological measures of posttraumatic stress symptoms questionnaire score (PCL) and the depression scale (CESD) at 6 months post-injury. (**A**) The significant white-matter tract is shown in red. (**B**–**D**) Scatter plots with post hoc correlation results. The mTBI group had a significant positive association (red dots and solid line), while the control group did not (blue dots and dashed line). Abbreviations: p_int_ = significance level of the group interaction term in Equation (3); R^2^ = overall coefficient of determination for goodness of fit for Model 3; *p*_mTBI_ = significance level of the association for the mTBI group; r_mTBI_ = correlation coefficient for the mTBI group; *p*_con_ = significance level of the association for the control group; r_con_ = correlation coefficient for the control group. Bold numbers indicate statistical significance with *p* < 0.05.

The associations between the early DTI metrics and later pain-related measures had significant group differences in three cerebral white-matter tracts, including the left insula, right sagittal stratum, and left superior longitudinal fasciculus (Table 3 and Figure 3A, Figure 4A and Figure 5A). While the mTBI group had significant associations indicating the predictivity of the early DTI metrics and later pain and psychological outcomes (Table 3), the control group did not have any significant associations (*p*_con_ > 0.05, Table 3). Overall, PCL and CES-D are the most sensitive psychological measures to early microstructural changes after mTBI. The directionality of the associations can be evaluated by the scatter plots in Figure 3, Figure 4 and Figure 5, where worse psychological scores in the mTBI group were associated with and could be predicted by elevated diffusivities (MD and RD) and reduced tissue micro-organization (FA).

## 4. Discussion

In this study, we performed DTI to detect white-matter microstructural changes in mTBI subjects and their relationships with headache and risk factors for the development of persistent PTH. AD was found to be significantly lower in the forceps major in the subacute phase (1 month) of mTBI. The forceps major connects the prefrontal and fronto-orbital regions, and its impairment may suggest impaired perceptual, cognitive, and motor-related functions following injury [71,72]. Lower AD in the mTBI participants is supported by previous studies [39]. For patients with TBI, the previous literature using automated fiber quantification has also identified subtle reductions in RD in this region [73]. In addition, studies on migraine have found lower AD in forceps minor among migraineurs, as well as in the white-matter fiber bundle connecting the mPFC and amygdala, which is associated with pain chronification [45,46].

Our results suggest that the early DTI metrics were predictive of later pain- and psychological-related measures. For the quantitative sensory tests, decreased white-matter organization predicted increased endogenous facilitation of pain and decreased endogenous inhibition of pain. Additionally, decreased parallel water diffusion (i.e., AD along the axonal orientation) predicted increased pain sensitization of the head area. The early DTI metrics were also sensitive to psychological measures. Decreased white-matter organization was associated with greater post-traumatic stress and depression symptoms. On the other hand, increased diffusivities were associated with later post-traumatic stress, pain catastrophizing, and depression symptoms. Increased diffusivities were also associated with greater self-reported headaches. The predictivity of the DTI metrics had significant group differences in the psychological measures and PPT, in which the control group did not exhibit such relationships.

The direction of DTI metrics changes may have clinical implications. For example, “an abnormal increase in MD may indicate destruction of the tissue microarchitectures such as axonal beading, cellular swelling, demyelination, or brain edema. An abnormal increase in RD indicates structural destruction perpendicular to the axons such as demyelination, while an abnormal increase in AD indicates structural destruction parallel to the axons such as destruction of cytoskeletons. An abnormal decrease in FA may indicate disorganization axons or demyelination” [44]. In this study, the overall directionalities of the associations to predict worse QST, psychological, and clinical measures at 6 months post-injury were decreased anisotropy (FA) and increased diffusivities (i.e., MD, AD, and RD). Such directionalities may indicate degraded white-matter organizations with dispersed and impaired ordering of axonal fibers and increased intra- and extra-axonal space, enabling freedom of water diffusion. Our results, which demonstrated that higher water mean diffusion is linked to lower clinical outcomes, are consistent with the previous literature. One study showed that mTBI patients with poor clinical tests had elevated MD in the bilateral superior longitudinal fasciculus 4 months after injury [42]. Increased MD in the bilateral cingulum angular bundles were also associated with high headache frequency in persistent PTH patients [47].

From our results, the most sensitive white-matter tracts appeared to be the left insula, right sagittal stratum, and left superior longitudinal fasciculus. Insula is an essential component of the pain matrix for pain perception with extensive structural connections to the prefrontal, parietal, and central cingulate gyri [74,75,76]. Striatum, composed by caudate, putamen, and ventral striatum, receives afferents from the cortex, midbrain, and thalamus and deliverers signal to the basal ganglia [77,78]. The neural activity in striatum is mainly in response to movements and rewards [79,80]. The superior longitudinal fasciculus (SLF), which connects the frontal, parietal, and occipital lobes [39], is highly relevant to prefrontal cortical pain modulation [40,41].

There are several limitations in this study. The primary constraint was the modest sample sizes, which may minimize the capability of detecting small effect sizes, limit generalizability, restrict statistical analyses from performing multiple comparisons, and increase the risk of type II errors [81]. The limitation came from the general challenges in longitudinal recruitment of TBI patients with comprehensive quantitative pain-related measures. Nevertheless, the effect sizes, statistical evaluators independent from sample sizes, of the associations (i.e., r, the correlation coefficients) in Table 2 and Table 3 are strong and are larger than 0.6. Meanwhile, our findings are consistent with previous studies that have reported similar group differences in diffusivity or significant associations between diffusion measures and pain metrics [39,42,73]. We believe that with multiple steps of direct association analyses and interaction analyses, our results can still provide the community with useful knowledge and effect sizes for future large-scale study designs. Furthermore, the diffusion tensor assumption has its limitations, as it uses a Gaussian distribution to summarize overall water diffusion behavior within an imaging voxel comprising multiple tissue types, crossing fibers, and diffusion compartments. This approach may not accurately reflect the true complexity of brain tissue. We, therefore, had limited our analyses to the center of the white-matter tracts (skeletonized white-matter ROIs), where the diffusion tensor assumption is less violated. Future diffusion MRI could focus on biophysical modeling, such as neurite orientation dispersion and density imaging [82] or kurtosis-based white-matter tract integrity imaging [83], to produce more biologically specific diffusion metrics. In summary, while our study provides meaningful inputs to the understanding of association between mTBI and pain in PTH, future research should focus on larger sample sizes, long-term follow-up for studying chronicity and progression of PTH, and more advanced imaging techniques to enhance the robustness and applicability of the findings.

There have been very few longitudinal studies systemically examining the pain-related white-matter changes after mTBI [50]. Here, we investigated the associations between white-matter integrity and the comprehensive measures of pain-related sensory, psychological, and clinical outcomes at the subacute to chronic stage. Using our sophisticated image-processing pipelines and analysis with an interaction model, we demonstrated that the DTI metrics in white-matter are predictive of pain and psychological measures in mTBI. Despite the limitations, this study provides critical insights and effect sizes, paving the way for future studies. One key implication from our study is that pain threshold measurements may be implemented as a standard add-on for mTBI participants to better understand pain-related changes over time. Additionally, given the observed associations between diffusion imaging and pain, psychological, and clinical measures, diffusion imaging could serve as a non-invasive and pain-free method to predict chronic, persistent pain in individuals with mTBI. Lastly, considering all four DTI metrics provides a more comprehensive interpretation of the microstructural mechanisms underpinning mTBI and PTH, providing potential therapeutic targets.

## 5. Conclusions

Our longitudinal study demonstrated that the DTI metrics at 1 month post-injury are sensitive to mTBI and are predictive of pain and psychological measures in the chronic stage of mTBI at six-months post-injury.

## Figures and Tables

**Table 1:** Demographic, pain, psychological, and clinical measurements of the participants.

	Time Point	mTBI	Control	*p*-Value
Demographics				
Sample size (*n*)		12	10	N/A
Age (year)		34.00 (8.09)	31.30 (8.38)	0.45
Sex (male:female)		9:3	5:5	0.44 *
Quantitative sensory tests (QST)				
TS	1 mo	12.13 (17.66)	15.25 (16.97)	0.68
	6 mo	9.8 (18.48)	12.22 (5.42)	0.71
PPT	1 mo	293.95 (149.38)	344.61 (115.00)	0.39
	6 mo	333.82 (182.03)	411.80 (115.37)	0.29
CPM	1 mo	13.27 (18.17)	23.02 (18.90)	0.23
	6 mo	49.39 (91.00)	21.26 (17.45)	0.40
Psychological measures				
PCS	1 mo	13.17 (11.33)	13.20 (11.55)	0.99
	6 mo	14.60 (10.32)	23.11 (17.59)	0.21
PCL	1 mo	23.08 (18.74)	8.10 (6.06)	**0.03**
	6 mo	24.00 (22.42)	9.00 (9.25)	0.08
CES-D	1 mo	18.42 (13.10)	8.30 (8.21)	**0.04**
	6 mo	16.90 (13.39)	8.56 (8.32)	0.13
Clinical measures				
HA	1 mo	6.00 (2.68)	0.70 (2.21)	**0.0001**
	6 mo	5.11 (3.69)	0.78 (2.33)	**0.01**
MPQ	1 mo	10.25 (6.05)	0.10 (0.32)	**0.0001**
	6 mo	7.60 (5.50)	1.22 (3.67)	**0.01**
TBI-QOL	1 mo	22.33 (5.65)	1.00 (3.16)	**0.00001**
	6 mo	23.30 (13.05)	2.33 (4.64)	**0.001**
DVRS pain rating at MRI scan	1 mo	3.17 (2.33)	0.00 (0.00)	**0.001**

Data are presented as mean (standard deviation) or male: female. Two tailed *t*-tests were used unless noted otherwise. Bold numbers indicate statistical significance with *p* < 0.05. * denotes Pearson’s Chi-square tests used for sex.

**Table 2:** Significant associations between the DTI metrics of the mTBI participants at 1 month post-injury and pain-related measures at 6 months post-injury.

	DTI Metrics	*p*	r	R^2^
Quantitative sensory tests (QST)				
Right sagittal stratum				
TS	FA	0.01	−0.60	0.80
Left anterior thalamic radiation				
PPT	AD	0.04	0.23	0.87
Left corticospinal tract				
PPT	AD	0.01	0.35	0.94
Left insula				
PPT	AD	0.02	0.13	0.90
Left superior longitudinal fasciculus				
CPM	FA	0.02	0.83	0.84
Psychological measures				
Right sagittal stratum				
PCL	FA	0.02	−0.60	0.75
PCL	RD	0.04	0.66	0.67
CES-D	FA	0.03	−0.61	0.75
CES-D	RD	0.03	0.69	0.74
Left insular				
PCS	MD	0.03	0.68	0.70
Left Corticospinal tract				
PCS	AD	0.04	0.69	0.64
Left superior longitudinal fasciculus				
PCL	MD	0.03	0.73	0.71
PCL	RD	0.03	0.77	0.71
CES-D	MD	0.03	0.85	0.73
CES-D	RD	0.04	0.78	0.72
Clinical measures				
Left Corticospinal tract				
MPQ	RD	0.01	0.67	0.84

Statical Model 2 (Equation (2)) was used with age, sex, and the DVRS scores as covariates. Abbreviations: r = correlation coefficient; R^2^ = coefficient of determination for goodness of fit.

**Table 3:** Significant group differences in the associations between the DTI metrics at 1 month post-injury and pain-related measures at 6 months post-injury.

		Model 3		mTBI _(post hoc)_		Control _(post hoc)_	
	DTI Metrics	*p*_int_	R^2^	*p*_mTBI_	r_mTBI_	*p*_con_	r_con_
Quantitative sensory tests (QST)							
Left insula							
PPT	AD	0.04	0.53	**0.02**	**0.13**	0.35	−0.10
Psychological measures							
Right sagittal stratum							
PCL	FA	0.00 *	0.53	**0.02**	**−0.60**	0.8	−0.04
PCL	RD	0.01	0.55	**0.04**	**0.66**	0.65	−0.04
CES-D	FA	0.01	0.48	**0.03**	**−0.61**	0.19	0.14
CES-D	RD	0.01	0.54	**0.03**	**0.69**	0.18	−0.15
Left superior longitudinal fasciculus							
PCL	MD	0.03	0.52	**0.03**	**0.73**	0.27	−0.26
CES-D	MD	0.02	0.57	**0.03**	**0.85**	0.45	0.04
CES-D	RD	0.05	0.51	**0.04**	**0.78**	0.20	−0.19

Statical Model 3 (Equation (3)) with group interaction term was used with age, sex, and the DVRS scores as covariates. Post hoc associations using Model 2 (Equation (2)) were performed for individual groups (i.e., the mTBI and control groups). Abbreviations: *p*_int_ = significance level of the group interaction term in Equation (3); *p*_mTBI_ *=* significance level of the association between DTI and pain-related measures for the mTBI group; r_mTBI_ = correlation coefficient for the mTBI group; *p*_con_ *=* significance level of the association between DTI and pain-related measures for the control group; r_con_ = correlation coefficient for the control group. Bold numbers indicate statistical significance with *p* < 0.05. * denotes *p_int_* = 0.0044.

## Data Availability

De-identified data from this study will be made publicly available in (FITBIR). Analytic code used to conduct the analyses will be made available in (GITHUB).

## Supplementary Materials

The following supporting information can be downloaded at https://www.mdpi.com/article/10.3390/diagnostics15050642/s1: Table S1: Kolmogorov-Smirnov Normality Test: *p*-values for Patient and Control Groups Across QST, Psychological, and Clinical Measures (6 month); Table S2: Kolmogorov-Smirnov Normality Test: *p*-values for Patient and Control Groups Across DTI metrics.

## Abbreviations

The following abbreviations are used in this manuscript:

DTI	Diffusion tensor imaging
mTBI	Mild traumatic brain injury
PTH	Post-traumatic headache
PPT	Pressure pain thresholds
CPM	Conditioned pain modulation
MRI	Magnetic resonance imaging
SWI	Susceptibility-weighted imaging
FA	Fractional anisotropy
MD	Mean diffusivity
RD	Radial diffusivity
AD	Axial diffusivity
MMSE	Mini Mental Status Examination
QST	Quantitative sensory tests
TS	Temporal summation
CPM	Conditioned pain modulation
HA	Headache intensity
MPQ	McGill Pain Questionnaire
PRI	Pain rating index
DVPRS	Defense and Veterans Pain Rating Scale
TBI-QOL	TBI Quality of Life
PTSD	Post-Traumatic Stress Disorder
PCS	Pain Catastrophizing Scale
CES-D	Center for Epidemiological Studies-Depression Scale
IRSPGR	Inversion recovery-prepared spoiled gradient-echo
GRE	Gradient-echo sequence
FSL	FMRIB Software Library
MNI	Montreal Neurological Institute
ROIs	Regions-of-interest
TBBS	Tract-based spatial statics
*p*	Significance level
JHU	Johns Hopkins University
mo	Month
PCL	Post-traumatic stress symptoms questionnaire
r	Correlation coefficient
R^2^	Coefficient of determination for goodness of fit
*p*_int_	Significance level of the group interaction term
*p*_mTBI_	Significance level of the association between DTI and pain-related measures for the mTBI group
r_mTBI_	Correlation coefficient for the mTBI group
*p*_con_	Significance level of the association between DTI and pain-related measures for the control group
r_con_	Correlation coefficient for the control group
SLF	Superior longitudinal fasciculus
