# Supplementary material for: Pain-Related White-Matter Changes Following Mild Traumatic Brain Injury: A Longitudinal Diffusion Tensor Imaging Pilot Study
Source: Diagnostics (Basel). 2025 Mar 6;15(5):642. doi: 10.3390/diagnostics15050642 (PMC11898438; doi:10.3390/diagnostics15050642)
Supplement: Supplementary file 1 [file diagnostics-15-00642-s001.zip › diagnostics-3460294-supplementary.pdf]

Table S1. Kolmogorov-Smirnov Normality Test: p-values for Patient and Control Groups Across QST, Psychological, and Clinical Measures (6 month).

	mTBI(n=12)	Control (n=10)
**Quantitative sensory tests (QST)**		
TS	0.13	0.73
PPT	0.62	0.68
CPM	0.91	0.91
**Psychological measures**		
PCS	0.92	0.99
PCL	0.47	0.14
CES-D	0.89	0.69
**Clinical measures**		
HA	0.98	0.008
MPQ	0.52	0.008
TBI-QOL	0.92	0.02

A p-value larger than 0.05 indicates that the data comes from a standard normal distribution.

Table S2. Kolmogorov-Smirnov Normality Test: p-values for Patient and Control Groups Across DTI metrics.

	mTBI (n=12)				Control (n=10)			
White matter tracts	FA	MD	AD	RD	FA	MD	AD	RD
Insular Cortex L	0.09	0.97	0.92	0.96	0.95	0.85	0.90	0.98
Insular Cortex R	0.43	0.88	0.73	0.91	0.95	0.96	0.41	0.36
Cingulum cingulate gyrus L	0.78	0.78	0.89	0.99	0.87	0.95	0.91	0.81
Cingulum cingulate gyrus R	0.61	0.98	0.89	0.99	0.65	0.97	0.91	0.73
Cingulum hippocampus L	0.93	0.77	0.99	0.76	0.81	0.74	0.72	0.45
Cingulum hippocampus R	0.63	0.43	0.64	0.70	0.80	0.96	0.68	0.69
Corticospinal tract L	0.97	0.99	1.00	1.00	0.77	0.89	0.80	0.73
Corticospinal tract R	0.89	0.63	0.59	0.92	0.69	0.39	0.72	0.93
Posterior thalamic radiation include optic radiation L	1.00	0.99	1.00	0.96	0.96	0.96	0.44	0.82
Posterior thalamic radiation include optic radiation R	0.67	0.89	0.92	0.85	0.48	0.92	0.34	0.82
Sagittal stratum include inferior longitidinal fasciculus inferior fronto occipital fasciculus L	0.35	0.89	0.83	0.52	0.24	0.89	0.97	1.00
Sagittal stratum include inferior longitidinal fasciculus inferior fronto occipital fasciculus R	0.77	0.53	0.79	0.50	0.53	0.89	0.75	0.52
Superior longitudinal fasciculus L	0.86	0.92	0.84	0.49	0.90	0.99	0.40	0.89
Superior longitudinal fasciculus R	0.51	0.17	0.73	0.22	0.94	0.93	0.95	0.65
Anterior thalamic radiation L	0.86	0.25	0.98	0.77	0.42	0.60	0.89	0.38

Anterior thalamic radiation R	0.97	0.41	0.89	0.95	0.98	0.41	0.74	0.28
Forceps major	1.00	0.88	0.87	0.90	0.99	0.93	0.95	1.00
Forceps minor	0.81	0.36	0.73	0.81	0.90	0.19	0.88	0.21

A p-value larger than 0.05 indicates that the data comes from a standard normal distribution.
